# Academical Proceedings, National and Foreign

**Published:** 1822-01

**Authors:** 


### ACADEMICAL PROCEEDINGS,

### NATIONAL AND FOREIGN.

---

### ROYAL SOCIETY OF LONDON.

*Nov.* 8.—The Society resumed its meetings after the long vacation, Sir H. Davy in the Chair, and numerous visitors present.

The minutes of the last meeting, in July, were read, and an account given of—I. A Paper by Mons. Wronsky on the Theory of the Earth. II. A Memoir by Mr. Charles Bell, proposing a new Classification of the Nerves, particularly of those of the respiratory organs: and III. A joint Essay by Messrs. Phillipps and Farraday on a new combination of Chlorine and Carbon.

Dr. Paris and Dr. Frank, having signed the obligation-book, were successively introduced to the President by Dr. Maton, and admitted fellows of the society.

The Croonian Lecture by Sir E. Home, Bart. one of the vice-presidents of the society, on the Adjustments of the Human Eye, was then read in part, and the remainder postponed till the next meeting. The author engaged the valuable services and assistance of Mr. Bauer in the new investigation he has undertaken of a subject which has been so frequently discussed. The microscopical observations of that gentleman lead him to assert, that, the marsupium in the eye of birds, which Sir Everard had on a former occasion contended to be muscular, is only membranous; and that the ciliari processes in the human eye are not muscular but vascular. M. Bauer has farther discovered lymphatics on each side of the arteries in the choroid coat, and Sir E. avers, that, both the marsupium in birds, and the choroid coat in the human eye, serve the purpose of secreting the nigrum pigmentum; which latter substance, Sir E. thinks, is nothing else than the colouring matter of blood rendered black in the act of secretion. The lecturer then goes on to discuss the question of the contractility of the iris, and refers to the several experiments of Mr. Dolland, Dr. Wells, Mr. Ware, and Dr. Roget.

*Nov.* 15.—A. Majendie, Esq. was admitted a fellow of the society.

The conclusion of the Croonian Lecture, by Sir E. Home, was read, containing the explanation offered by Mr. Dolland on the use of the marsupium in birds, and the geometrical dimensions of the retina in the eye of those animals, in which that organ differs from the retina in the human eye.

*Nov.* 22.—Sir H. Davy in the Chair. The minutes of the last meeting being read; Messrs. Coen and Hall were admitted fellows.

The President then rose and said, that it was his painful duty to propose to the society, the ejection of a fellow from among them. Mr. Charles Tweedie, having been declared a defaulter to a large amount by the heads of the department, it would reflect a disgrace on the Royal Society at large, if the name of that individual were suffered, any longer, to appear in the list of its members. He had, therefore, to propose that his name should be erased from the list; and, on the ballot-box having gone round, it appearing to be the unanimous opinion of the society that Mr. Charles Tweedie should be expelled, the President drew his pen across the name of that individual in the Statute-book, according to the usual forms on such occasions.

The reading of the Bakerian Lecture by Capt. Sabine, on the Dip of the Magnetic Needle, which had been partially read at the last meeting, was concluded. A letter was read from the astronomer-royal, Professor Pond, to Sir H. Davy, giving an account of a derangement which had taken place in the mural circle at the Observatory of Greenwich, and which ought to be made known to astronomers as soon as possible, with a view to correct the errors that have resulted from that derangement, in reference to the observations published since September 1819. The cause of the derangement and the means adopted to remedy it, are pointed out in the letter.

An Account of some Alvine Concretions, found in a young man who died at Clitheroe, was communicated by G. Children, Esq. from the Society for promoting Animal Chemistry. The conclusion of this paper was adjourned till the next meeting.

*Nov.* 30.—Anniversary meeting of the society, for the election of officers.

The Treasurer's account for the year preceding was read, from which it appeared, that the funds of the society were in a flourishing condition. The balance in hand on the current year's account, only, amounts to 297*l.* 10*s.* The number of new fellows admitted in 1820 appeared to be 45, that of foreign members 2.

Two of the Copleyan medals were afterwards awarded, by the

President and Council, to Mr. Herschel and Capt. Sabine. To the former for his mathematical papers, inserted in the last volume of the Philosophical Transactions, and for his two memoirs on the Polarization of Light and the Aberration of Spherical Surfaces,—and to the latter for his Observations on the Dip of the Magnetic Needle in the Arctic Regions. The President, in presenting the medals to the candidates, delivered a suitable eulogium on their labours, giving, at the same time, a clear and elegant analysis of their memoirs, agreeably to custom.

The society then proceeded to the election of new officers and council for the ensuing year, when the following gentlemen were elected.

*Of the old Council.*—Sir Humphry Davy, Bart.; W. T. Brande, Esq.; the Lord Bishop of Carlisle; Taylor Combe, Esq.; Davies Gilbert, Esq.; Charles Hatchett, Esq.; J. F. W. Herschel, Esq.; Sir Everard Home, Bart.; John Pond, Esq.; Wm. Hyde Wollaston, M.D.; Thomas Young, M.D.

*Of the new Council.*—The Earl of Aberdeen; Matthew Baillie, M.D.; John Barrow, Esq.; H. C. Brodie, Esq.; Wm. Hamilton, Esq.; James Ivory, Esq.; the Marquis of Lansdowne; Alexander Marcet, M.D.; Thomas Murdoch, Esq.; Sir Robert Seppings, Knt.

*And the Officers.*—President, Sir Humphry Davy, Bart., LL.D.—Treasurer, Davies Gilbert, Esq.—Secretaries, William Thomas Brande, Esq. and Taylor Combe, Esq.

After the election, the members of the society dined together, as usual, at the Crown and Anchor Tavern.

*Dec.* 6.—This meeting was principally occupied in reading the minutes of the anniversary meeting, and of the two discourses pronounced by Sir Humphry Davy on that occasion.

Dr. Cooke, the president of the Medico-Chirurgical Society, was elected a fellow, by one of the largest assemblage of fellows present at any ordinary meeting. The reading of the paper on certain alvine concretions was continued.

*Dec.* 13.—The paper on alvine concretions was concluded. The calculi were successively submitted to the action of cold water, boiling water, alcohol, caustic soda, and muriatic acid,—and were found to consist principally of the ammoniaco-magnesian phosphate, animal matter, vegetable fibre, and a peculiar kind of *farina.*

Dr. Ure, the professor of chemistry at Glasgow, was elected a fellow of the society.

A paper was read by Dr. Wollaston, on a catoptrical adjustment to a triple-glass telescope.

Sir Everard Home gave an account of a new species of rhinoceros found in Africa, the skull of which closely resembles that of the rhinoceros found in a fossil state in Siberia. The skull was brought home by that active missionary, Mr. Campbell. The animal feeds on grass and bushes, is not unfrequently met with, and is not gregarious. The long horn, which is placed on the nasal bone, measures 36 inches in length, and is 24 inches in circumference at its base.

*Dec.* 20.—The Bishop of Carlisle in the Chair. A paper was read from Sir H. Davy, the president, giving an account of some experiments made to prove that luminous electrical phenomena take place in vacuo.—The society then adjourned over the Christmas holydays, till the 8th of January, 1822.

☞ *Want of room obliges us to postpone the account of the meeting of other scientific and medical societies.*

*The department of* NECROLOGY *must also, for the same reason, be omitted.*

### Medical and Physical Intelligence.

